# The Presence of Mercury in the Tissues of Mallards (*Anas platyrhynchos* L.) from Włocławek Reservoir in Poland

**DOI:** 10.1007/s12011-016-0845-6

**Published:** 2016-09-21

**Authors:** Jerzy F. Żarski, Michał Skibniewski, Ewa Skibniewska, Tadeusz P. Żarski, Teresa Majdecka

**Affiliations:** 1 Department of Animals Environment Biology, Faculty of Animal Science, Warsaw University of Life Sciences – SGGW, Ciszewskiego 8, 02-786 Warsaw, Poland; 2 Department of Morphological Sciences, Faculty of Veterinary Medicine, Warsaw University of Life Sciences – SGGW, Nowoursynowska 159 C, 02-776 Warsaw, Poland; 3 Halina Konopacka Higher School of Physical Culture and Tourism, Andrzeja 1, 05-800 Pruszków, Poland

**Keywords:** Mercury, Mallard, Liver, Kidneys, Muscles

## Abstract

The study aimed at determining the degree of mercury contamination of mallards, game waterbirds migrating from the regions of the unknown degree of contamination and establishing whether the consumption of their meat comprises a hazard to human health in view of the binding norms concerning the mercury content in food products. The investigations were carried out on 30 mallards shot during the duck shooting season in which mercury concentrations in the muscles, liver, and kidneys were determined using the cold vapor atomic absorption spectrometry (CV-AAS) method. The mean Hg concentration in the investigated tissues in all birds studied amounted to 0.110, 0.154, and 0.122 mg kg^−1^ for the muscles, kidneys, and liver, respectively. The study indicated statistically significant (*p* ≤ 0.01) positive correlation between all of the organs examined. Animals were divided into two groups differing in both absolute values of Hg concentrations and those measured in individual tissues. In particular organs of birds representing the first group, the presence of highly significant correlation (*p* ≤ 0.01) was observed in all organs examined. In the second group, highly significant positive correlation between Hg concentrations in the liver and kidneys and highly significant negative dependence between the liver and muscles was noted. The examinations revealed that some birds must have come from regions of a high degree of mercury contamination.

## Introduction

Mercury is a metal that may enter the environment both by natural processes of the earth’s crust and as a result of human activity. Released to the biosphere, mercury undergoes complex transformations, circulating between the atmosphere, terrestrial systems, and the aquatic environment. Plants, animals, and humans are subject to exposure to its toxicity, as long as mercury remains in its biogeochemical cycle [1–4]. Three basic forms of mercury occur in the environment: metallic mercury (Hg^0^), inorganic mercury (mercurous, Hg_2_^2+^, and mercuric cations, Hg^2+^), and organic mercury compounds [2]. Metallic or elemental mercury prevails in the atmosphere [5]. Inorganic mercuric compounds include mercury salts used in numerous technological processes. They are also found in electrical cells, fungicides, and disinfectants [6]. Organic forms of mercury are compounds in which the element binds with at least one carbon atom through a covalent bond. Methylmercury is one of the most common forms of organic mercury. This compound has the ability to accumulate and biomagnify at each step of the trophic chain of the aquatic ecosystems [7–10]. The toxic effects of mercury observed in homoiotermic vertebrates affect mainly the nervous, urinary, and reproductive systems [10, 11]. The inorganic mercuric compounds tend to accumulate in the kidneys and liver, whereas methylmercury penetrates into all the tissues of the body, including the skeletal muscles, nervous system, as well as the structures of the common integument [3, 10].

Numerous species of aquatic birds have been used for decades as bioindicators of the pollution of their habitats. The mallard (*Anas platyrhynchos*) is one of the most commonly studied species within the group of bioindicators. Among the reasons, underlying its common use in environmental studies is the wide distribution of the wild duck, since it occurs in the nearly entire Palaearctic ecozone [10]. In addition, the mallard is an excellent object of studies due to large populations, apparent sexual dimorphism, and longevity, which enables observations of long-term exposures to selected pollutants [12]. Not without significance is the fact that the mallard is a game species, the muscle tissue of which is consumed by humans. Therefore, knowing the content of toxic metals in mallard’s body is important in terms of food safety. Despite a large number of reports on the studies of heavy metals in the tissues of mallards, the data on mercury levels in the organs of the mallards inhabiting the area of Poland and other European countries are relatively scarce [9, 10].

In terrestrial animals, mercury enters the body through the respiratory system, gastrointestinal tract, and through the skin [9, 13]. It should be stressed, however, that there are considerable differences in mercury absorption depending on its form. Metallic mercury and its inorganic compounds are absorbed to a limited extent. Methylmercury, which after the intake undergoes demethylation in the liver, is the main compound [14].

The aim of the study was to determine total mercury content in the muscle, liver, and kidneys of mallards inhabiting the Włocławek Reservoir, using samples obtained from individuals arriving from areas of unknown levels of mercury pollution, and to determine whether consumption of its meat represents a risk to human health in the light of the current standards on tolerable mercury content in food products.

## Material and Methods

**Fig. 1:**
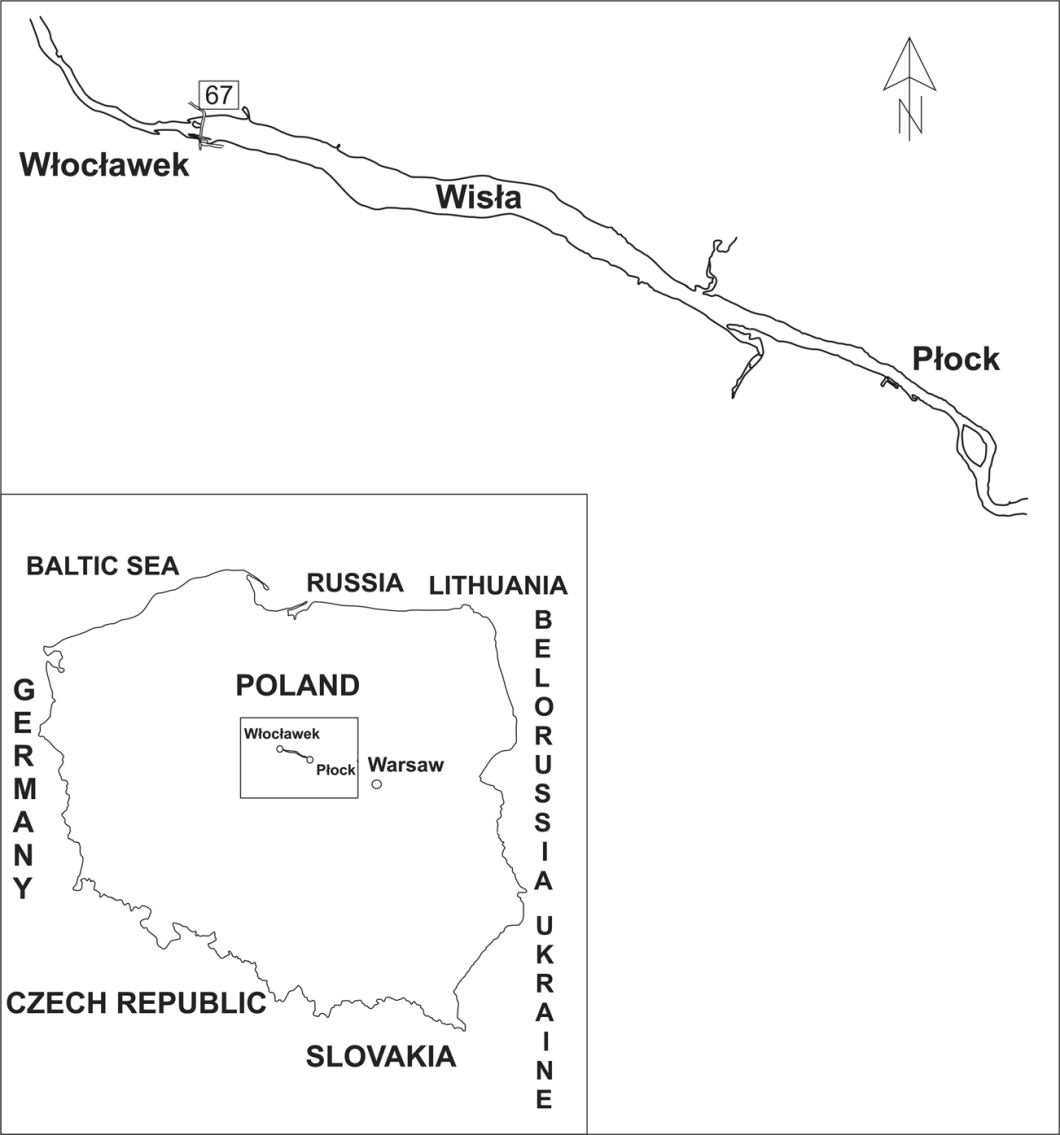
Sampling area in Poland, 67-road on a dam in Włocławek

The study was carried out using tissue samples collected from 30 mallards (*A. platyrhynchos*) of both sexes, with a body weights ranging from 800 to 1200 g, purchased from hunters during the autumn flights season. Only adult individuals were selected on the basis of plumage as each of them had large, angular black tip on the fifth greater secondary coverts. The ducks had been hunted during the 2013/2014 hunting season on the Włocławek Reservoir, which is Poland’s largest artificial lake. The reservoir was created in 1970 in the Middle Vistula flow by placing a dam on the river in Włocławek. It extends up to the city of Płock in the form of a ribbon lake with a length of 58 km and a width of 1.2 km (Fig. 1). The average water retention in the Reservoir is 5 days. Due to the fact that the lake is located in the middle course of the Vistula river, it tends to accumulate a considerable amount of organic matter.

Samples of the pectoral muscles (*musculus pectoralis major et musculus supracoracoideus*), liver, and kidneys were collected from each bird and stored frozen at −21 °C until analysis. Kidney samples were collected in a way to contain all of the three divisions (*divisio cranialis*, *media et caudalis*) of the organ. Before the analyses, the tissues and organs were thawed, and samples weighing not more than 300 mg were taken.

Mercury content in the samples was determined by cold vapor atomic absorption spectrometry (CV-AAS) using the automatic mercury analyzer AMA-254 (Altec, Prague, the Czech Republic). The apparatus was calibrated using a polarographically pure mercury standard in 2 % HNO_3_. The detection limits (LODs) obtained were 0.001 mg Hg kg^−1^. The method was validated for speciation analysis for mercury in BCR CRM 463 (tuna fish) reference material with the certified Hg concentration 2.85 mg kg^−1^. The result of certified reference material analysis was 2.49 mg kg^−1^ (recovery percentage 87.36).

The concentration of mercury in the samples was presented in milligram per 1 kg body weight (mg kg^−1^). Each measurement was replicated three times, and the result was expressed as the arithmetic mean of three measurements.

The resulting data were processed statistically using the Statistica 12™ package. Before analyses, the data were tested for normality with Shapiro-Wilk *W* test. Concentration of mercury was not normally distributed. Therefore, the non-parametric Mann-Whitney *U* test was used to check the significance of differences between groups. Relationships between the concentrations of mercury were calculated by using Spearman’s correlation coefficients at *p* ≤ 0.05 and *p* ≤ 0.01.

## Results

Concentrations of mercury in the liver, kidneys, and muscles of all birds studied are presented in Table 1. Taking into account the mean mercury concentrations in entire examined population, the highest values were noted in the liver. Lower levels of this metal were noted in the kidneys whereas the muscles were characterized by the lowest mean values of the mercury. However, the mean values did not differ statistically. We found statistically significant (*p* ≤ 0.01) positive correlation between all of the organs examined. On the base of detailed analysis, we divided studied animals into two groups differing in both absolute values of concentrations and those measured in individual tissues. The first group consisted of 23 individuals, whereas the second of 7 birds. Both were subjected to two separate statistical analyses. Concentrations of mercury in the tissues of individual animals from the first group as well as their statistical parameters are presented in Tables 2 and 3. Data relating to individuals from the second group are shown in Tables 4 and 5.

**Table 1:** Mercury concentrations in the organs of all mallards examined (*n* = 30) in milligram per kilogram of wet weight

Arithmetic mean—$\overline{\mathrm{X}}$		Liver	Kidneys	Muscles
		0.154	0.122	0.110
Standard deviations (SD)		0.145	0.092	0.196
Median		0.105	0.101	0.055
Q_25_		0.064	0.052	0.027
Q_75_		0.170	0.145	0.091
Value	Min	0.010	0.013	0.009
	Max	0.689	0.423	0.925
**Spearman’s correlation coefficients	Liver/….	–	0.834**	0.609**
	Muscles/…..	ns	0.702**	–

**Differences highly significant at *p* ≤ 0.01

*ns* non-significant

**Table 2:** Mercury concentrations in the organs of individual mallards from subpopulation I in milligram per kilogram of wet weight (*n* = 23, Σ < 0.600 mg kg^−1^)

Nr of sample	Liver	Kidneys	Muscles
1	0.037	0.046	0.023
2	0.057	0.052	0.027
4	0.112	0.050	0.020
5	0.094	0.194	0.069
6	0.061	0.034	0.028
7	0.086	0.124	0.050
8	0.167	0.064	0.040
9	0.102	0.118	0.061
10	0.170	0.145	0.099
11	0.098	0.094	0.039
12	0.064	0.035	0.019
13	0.018	0.013	0.009
14	0.091	0.090	0.087
17	0.010	0.079	0.037
18	0.203	0.132	0.109
19	0.109	0.097	0.091
20	0.167	0.116	0.051
21	0.195	0.164	0.083
22	0.126	0.130	0.068
23	0.065	0.058	0.024
24	0.061	0.046	0.029
27	0.060	0.027	0.017
28	0.144	0.127	0.058

**Table 3:** Mercury concentrations in the organs of mallards from subpopulation I (n = 23, Σ < 0.600 mg kg^−1^), in milligram per kilogram of wet weight

Arithmetic mean—$\overline{\mathrm{X}}$		Liver	Kidneys	Muscles
		0.100	0.088	0.049
Standard deviations (SD)		0.054	0.048	0.029
Median		0.094	0.090	0.009
Q_25_		0.061	0.046	0.024
Q_75_		0.144	0.127	0.069
Value	Min	0.010	0.013	0.009
	Max	0.203	0.194	0.109
**Spearman’s correlation coefficients	Liver/….	–	0.729**	0.718**
	Muscles/…..	ns	0.876**	–

**Differences highly significant at *p* ≤ 0.01

*ns* non-significant

**Table 4:** Mercury concentrations in the organs of individual mallards from subpopulation II in milligram per kilogram of wet weight (*n* = 7, Σ > 0.600 mg kg^−1^)

Nr of sample	Liver	Kidneys	Muscles
3	0.405	0.194	0.072
15	0.144	0.102	0.925
16	0.286	0.256	0.204
25	0.689	0.423	0.025
26	0.463	0.316	0.123
29	0.245	0.241	0.135
30	0.080	0.100	0.679

**Table 5:** Mercury concentrations in the organs of mallards from subpopulation II (*n* = 23, Σ > 0.600 mg kg^−1^), in milligram per kilogram of wet weight

Arithmetic mean—$\overline{\mathrm{X}}$		Liver	Kidneys	Muscles
		0.330	0.233	0.309
Standard deviations (SD)		0.208	0.116	0.349
Median		0.286	0.241	0.135
Q_25_		0.144	0.102	0.072
Q_75_		0.463	0.316	0.679
Value	Min	0.080	0.100	0.025
	Max	0.689	0.423	0.925
**Spearman’s correlation coefficients	Liver/….	–	0.893**	−0.893**
	Muscles/…..	ns	ns	–

**Differences highly significant at *p* ≤ 0.01

*ns* non-significant

In the analysis of correlation between the mercury levels in particular organs in the first group, the presence of highly significant dependence (*p* ≤ 0.01) was observed in all organs examined. In the second group, we noted highly significant positive correlation between mercury concentrations in the liver and kidneys and highly significant negative dependence between liver and muscles.

## Discussion

The mallard (*A. platyrhynchos*) is one of the most common aquatic avian species, which also represents a common object of many environmental studies. The world population of this duck is estimated to more than 28 million. Mallard population in the Europe alone is 7.5 million [15–17]. It is a migratory species. The mallards nest in moderate climate zones and, in colder seasons, migrate to wintering areas of milder climate. Changes in the habitats caused by anthropogenic activity have resulted in alterations in the life history of the species; some mallard populations in certain parts of the Europe refrain from seasonal migrations. This has been observed in large cities and urban areas that provide continuous availability of food [17–19]. Rural and other non-urban areas are mainly home to migrating populations, which are exposed to hunting. In Poland, according to current regulations, the season for hunting wild ducks extends from 15 August to 21 December [20]. The peak hunting yields were recorded in the mid-1990s, when the number of shot mallards might have reached 150,000 in a waterfowl hunting season. With the onset of the 21st century, these numbers slightly decreased, e.g., there were more than 115,000 wild ducks hunted in the season 2003/2004. Over the recent years, the number of hunted mallards in Poland has remained at a level of 100,000 birds [21]. The meat of these birds is highly valued by the consumers. There is no reliable data at hand as to how much mallard meat is consumed in Poland; however, if we consider the total number of hunted ducks, we may presume that several tones of wild duck meat, with the prominent, most popular mallard, reach the tables of the consumers in Poland every year.

The European Union food safety regulations deal with the mercury levels in fish and other seafood organisms only, with an assumption that the remaining food groups do not pose a threat to human health. It has been concluded that the maximum tolerable mercury level in fish meat is 0.5 μg g^−1^ of wet tissue weight [3, 22]. It should be noted, however, that waterfowl species dwell in habitats where toxic metals often reach high concentrations. Analyses carried out so far have usually focused on lead contained in organs and tissues of game species, since the metal is a component of hunting ammunition [23, 24]. Mercury receives much less attention. In aquatic ecosystems, toxic metals accumulate mainly in the bottom sediments, where they can be transformed into much more toxic forms [25]. Mercury, for example, may be transformed to methylmercury, and so can be ingested by waterfowl [26]. After ingestion, methylmercury is transformed to less toxic inorganic mercuric compounds. The process of demethylation occurs in the liver; hence, a large part of the heavy metal contained in this organ is inorganic mercury, which is next removed from the organism with the bile [9, 14].

The average levels of mercury measured in organs of the all mallards studied were similar and ranged from 0.110 mg kg^−1^, in the muscles, to 0.154 mg kg^−1^, in the liver. Our results are not consistent with the data of other authors who reported that mercury distribution among the tissues deviates considerably. They emphasize a great variability in the accumulation of mercury in different parenchymatous organs [27, 28]. In mammals, the kidneys are main organs of accumulation—they reveal areas marked by elevated concentrations of this element. Puls [29] noticed that mercury concentrations in the cortical part of the kidney may by 5 to 10 times higher compared to its medullary portion. In aquatic birds, considerable differences in mercury levels have been found between the muscle tissue and liver/kidneys. The latter organs exhibited significantly higher mercury levels; unlike in mammals, however, the differences between them were non-significant [9, 30]. The average concentration of mercury in the muscles, liver, and kidneys of mallards from the northwestern part of Poland was, respectively, 0.002, 0.380, and 0.380 mg kg^−1^ of wet weight [9]. The distribution of our results was similar to those reported by Binkowski et al. [10]. These authors observed that the median values of mercury concentrations in avian muscles and internal organs were similar. They also found that the medians of mercury concentrations from different muscle units located in various parts of the body did not differed considerably, with values ranging from 0.021 to 0.028 mg kg^−1^ wet weight.

**Fig. 2:**
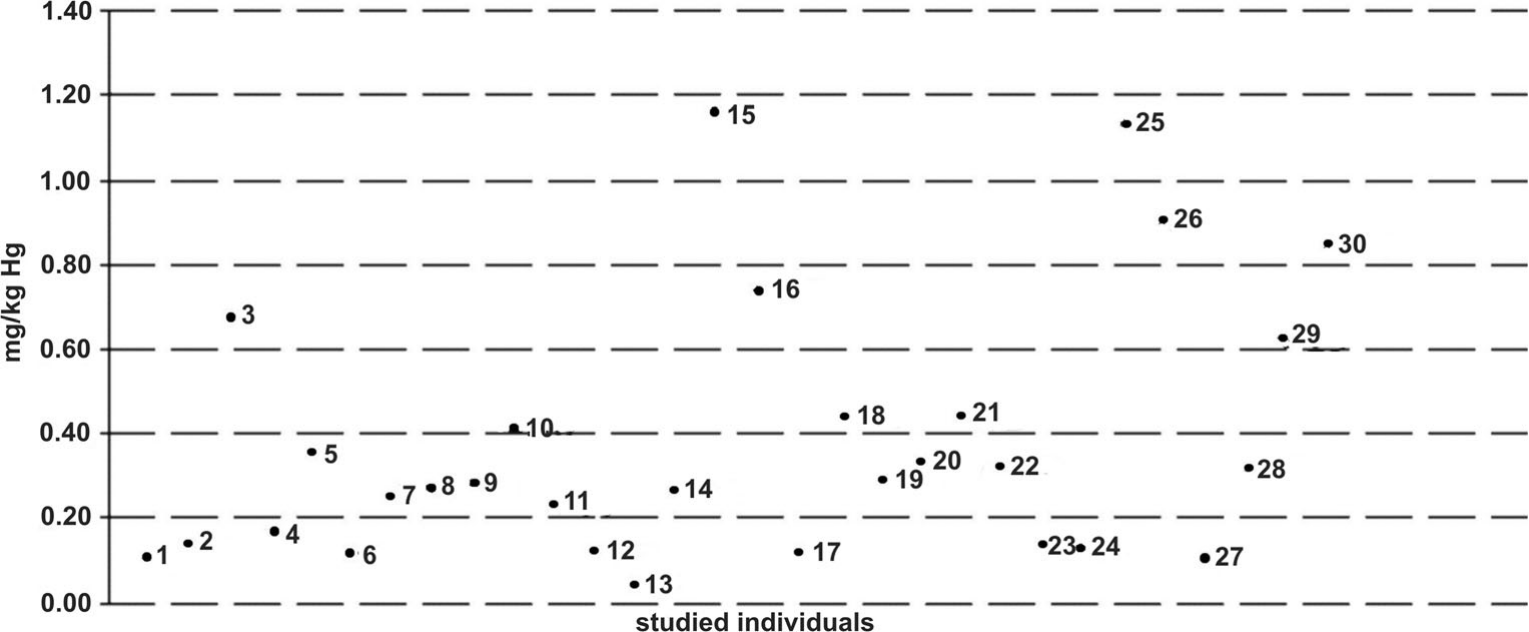
Sum of mercury concentrations in the liver, kidneys, and muscles of particular birds in the investigated population of mallards (where points 1–30 are the sum of values of mercury concentration in the liver, kidneys, and muscles in milligram per kilogram in particular investigated mallards)

Detailed analysis of the data clearly demonstrates that most summary concentrations (Fig. 2) in the studied mallard population ranged between 0.04 and 0.600 mg kg^−1^. This pertains to 23 birds, whereas the other 7 ducks represent a subpopulation differing in both absolute values of concentrations and those measured in individual tissues. Both naturally emerging subpopulations were subjected to two separate statistical analyses. As soon as the birds had been assigned to one of these two groups, the data equalized, as is evidenced by a decrease in the standard deviations both in the group formed basing on the lower total tissue mercury concentrations and in the group showing the higher concentrations. Mercury muscle concentrations in the group of 23 birds were nearly twice as low as those measured in the kidneys or the liver, which would confirm the fact that these two parenchymal organs play a major role in mercury accumulation and its removal from the body [28, 31]. The relatively high standard deviations result from the fact that the studied population was not homogeneous in terms of age, a factor which—besides the environmental impact—is well associated with the mercury accumulation levels. Namely, mercury tissue concentration increases with age, which results from the processes of accumulation predominating over the processes of removal from the animal body [9, 10, 32].

The pattern of mercury concentrations observed in the group of seven ducks (separated basing on the high total Hg levels in the studied tissues and organs) was surprising. Namely, the group included two individuals with very significantly higher muscle mercury concentrations, nearly 20 times higher than the average of the first group (23 ducks) and more than 10 times higher compared to the other individuals from their group.

This fact is all the more surprising that the mercury concentration in the liver and kidneys was in these two birds relatively low, and there could be no linkage with such a significant contamination of the muscles. This phenomenon is difficult to explain. High concentrations of mercury in the muscles of these individuals could be the result of absorption via the transdermal rather than gastrointestinal route [9, 13, 28, 33–35]. This would indicate that these individuals have come from a habitat where water bodies or soils were heavily polluted with mercury. In the remaining five mallards, the liver and kidney mercury levels were more than three times higher than in birds in the subpopulation I, which implies that they also came from areas of an elevated mercury pollution, where they had to feed on mercury-contaminated aquatic and terrestrial plants and animals living in water bodies.

## Conclusions

Analyzing the average mercury content in the tissues and organs of all the studied mallards, it should be stated explicitly that mercury levels in the tissues used as human food were relatively high. It seems, however—taking into account the share of meat of wild ducks in the diet—that its consumption should not cause violation of the WHO [36] recommendations that the provisional tolerable weekly intake (PTWI) of this element with food should not exceed 5 μg kg^−1^ body weight, of which methylmercury may not represent more than 1.6 μg kg^−1^ body weight. However, if we consider mean mercury concentration in the muscles of mallards from the second group, the estimated mercury intake resulting from the consumption of a 100 g portion of meat shows that a single meal covers approximately 9 % of PTWI for a person with body weight 70 kg. Therefore, the groups of risk may include only hunters, as they may more frequently consume mallard meat, as well as pregnant women and young children, due to the strong genotoxic and teratogenic effects of mercury and the risk that the toxic metal may damage the central nervous system. The authors are of the opinion that the meat of aquatic birds consumed by humans should be included in the system of mercury content monitoring supported by relevant regulations, as is the case of fish meat and seafood products.
